# Significant fibrosis assessed by liver biopsy among Chinese bariatric surgery patients: A prospective cross-sectional study

**DOI:** 10.3389/fendo.2023.1090598

**Published:** 2023-01-30

**Authors:** Yongsheng Huang, Shiliang Dong, Cunchuan Wang, Zhiyong Dong, Wenhui Chen

**Affiliations:** ^1^ Department of Gastrointestinal Surgery, The First People’s Hospital of Zunyi and Third Affiliated Hospital of Zunyi Medical University, Zunyi, Guizhou, China; ^2^ Department of Metabolic and Bariatric Surgery, The First Affiliated Hospital of Jinan University, Guangzhou, China

**Keywords:** non-alcoholic fatty liver disease, non-alcoholic steatohepatitis, fibrosis, bariatric surgery, obesity

## Abstract

**Background:**

Fibrosis stages affect clinical prognoses related to nonalcoholic fatty liver disease (NAFLD). However, data on the prevalence and clinical features of significant fibrosis are scarce in Chinese bariatric surgery patients. We aimed to investigate the prevalence of significant fibrosis in bariatric surgery patients and to identify its predictors.

**Methods:**

We prospectively enrolled the patients performing intra-operative liver biopsies during bariatric surgery from a bariatric surgery center in a university hospital between May 2020 and January 2022. Anthropometric characteristics, co-morbidities, laboratory data and pathology reports were collected and analyzed. The performance of non‐invasive models was evaluated.

**Results:**

Of 373 patients, 68.9%% had non-alcoholic steatohepatitis (NASH) and 60.9% exhibited fibrosis. Significant fibrosis was present in 9.1% of patients, advanced fibrosis in 4.0%, and cirrhosis in 1.6%. Multivariate logistic regression showed that increasing age (odds ratio [OR], 1.06; p=0.003), presence of diabetes (OR, 2.62; p=0.019), elevated c- peptide (OR, 1.26; p=0.025) and elevated aspartate aminotransferase (AST) (OR, 1.02; p=0.004) were independent predictors of significant fibrosis. The non-invasive models, AST to Platelet ratio (APRI), Fibrosis‐4 (FIB-4), and Hepamet fibrosis scores (HFS) provided greater accuracy for predicting significant fibrosis, compared to the NAFLD Fibrosis Score (NFS) and BARD score.

**Conclusion:**

More than two-thirds of bariatric surgery patients had NASH and the prevalence of significant fibrosis was high. Elevated levels of AST and c- peptide, advanced age and diabetes indicated a higher risk of significant fibrosis. Non-invasive models, APRI, FIB-4 and HFS can be used to identify significant liver fibrosis in bariatric surgery patients.

## Introduction

Nonalcoholic fatty liver disease (NAFLD), now known as metabolic-associated fatty liver disease (MAFLD), has become the most common cause of chronic liver disease worldwide (1, 2). Epidemiological research estimates a 25% prevalence in the general population, rising to 90% in patients undergoing bariatric surgery (3, 4). Some risk factors, including obesity, diabetes, hypertension, hyperlipidemia and metabolic syndrome are established indicators of NAFLD development (5). Thus, it is anticipated that as the prevalence of obesity and diabetes increases, so will that of NAFLD. Non-alcoholic steatohepatitis (NASH) is the active form of NAFLD. It is characterized by hepatocyte ballooning and lobular necroinflammation (which can occur with or without fibrosis) and may silently progress towards cirrhosis, end-stage liver disease, and even hepatocellular carcinoma (6, 7).

A strong correlation has been demonstrated between the degree of fibrosis and liver-specific morbidity and overall mortality in NAFLD patients (8). Furthermore, patients with significant fibrosis are most likely to experience complications and further progression of the hepatic disease (9). Unfortunately, most patients with fibrosis are asymptomatic and have normal transaminases. Thus, we need to detect risk factors for liver fibrosis, especially significant fibrosis, because distinguishing between NAFLD with or without significant fibrosis has important clinical significance for determining the prognosis (10, 11). Abdominal ultrasound is effective in detecting fatty liver but not liver fibrosis. To date, histologic evaluation of the liver biopsy remains the gold standard for diagnosing NASH and assessing the stage of fibrosis (6, 12). Nevertheless, liver biopsy is not a routine procedure due to its invasiveness, high costs, sampling variability and various potential complications. There are several non-invasive scoring systems specifically designed to identify the presence of advanced fibrosis which include: the aspartate aminotransferase (AST)-to-platelet ratio index (APRI) (13), BARD scoring system (14), NAFLD Fibrosis Score (NFS) (15) and Fibrosis-4(FIB-4) score (16). Some studies have shown that these non-invasive scoring systems were assessed to detect advanced fibrosis in morbid obesity or diabetes, but the application of these scores was from white and non-Asian populations (17, 18). Little is known about the reliability of non-invasive scoring systems to detect significant liver fibrosis in Chinese bariatric patients. (Reviewer #1). In addition, these scoring systems were developed using data from viral hepatitis patients and have yet to be validated for Chinese bariatric surgery patients. Thus, we determined to test the hypothesis that these algorithms were able to identify significant liver fibrosis among bariatric surgery patients.

Currently, research data reporting on the prevalence and clinical characteristics of fibrosis mainly originate from Western countries (3, 19, 20). However, there has yet to be a study that specifically evaluates the prevalence of significant fibrosis (and its associated predictors) in the Chinese population. In fact, China is one of the countries with the largest population of obesity, and the obesity phenotype is mainly moderate obesity (21). In addition, given that Chinese eating habits and lifestyles are particularly distinctive compared with those of other nationalities, the prevalence of significant fibrosis may vary considerably compared with data published to date. Determining potential risk factors for significant fibrosis may help clinicians perform risk stratification of bariatric surgery patients with NAFLD, facilitating early identification of high-risk populations. Thus, this study aimed to evaluate prevalence and clinical predictors of hepatic fibrosis (confirmed by biopsy) experienced by Chinese bariatric surgery patients. In addition, we look to validate the reliability of the aforementioned, non-invasive fibrosis scoring algorithms.

## Materials and methods

### Study population

This is a prospective, observational study of a cohort of Chinese bariatric surgery patients. In this study, patients were recruited from a bariatric surgery center in a tertiary university hospital during the period May 2020-January 2022. Then inclusion criteria for this study were as follows: (1) patients who met metabolic surgery standard: body mass index (BMI) ≥ 32.5 kg/m^2^ or BMI ≥ 27.5 kg/m^2^ with poor weight loss by medications or lifestyle modification and with at least two components of metabolic syndrome or with comorbidities (22); (2) patients who had consented to a trans-operative liver biopsy. The exclusion criteria were:(1) patient had any history of alcoholism (average daily consumption of alcohol of 30 g/day for men and 20 g/day for women); (2) patients tested positive for viral hepatitis (B or C); (3) patients had incomplete pathology reports. (4) patients with diabetes take insulin treatment; (5) patients underwent preoperative weight loss or very low-calorie diets. (Reviewer #3) The study was approved by our hospital ethics committee (2019-024). Written informed consent was obtained from each participant or legal representatives before bariatric surgery.

### Clinical and laboratory data

Clinical and laboratory data was sourced from a prospectively collected database (KY-2020-021). Demographic data (gender, age), anthropometric data (weight, BMI, waist circumference, hip circumference, waist to hip ratio) and the presence of co-morbidity (Metabolic syndrome, hypertension, type-2 diabetes mellitus(T2DM)) were analyzed. BMI was calculated by dividing body weight by the square of body height. Metabolic syndrome was defined as the presence of at least 3 of the 5 following criteria (23): (1) abdominal obesity (waist circumference ≥ 90 cm in man and ≥ 80 cm in women); (2) blood pressure ≥130/85 mmHg or taking antihypertensive drug; (2) serum triglycerides ≥1.7 mmol/L, or taking lipid-lowering drugs; (4) serum high-density lipoprotein cholesterol (HDL-c) <1.0 mmol/L for man and <1.3 mmol/L for women, or drug treatment for reduced HDL-c; (5) fasting plasma glucose (FPG) ≥5.6 mmol/L), or drug treatment for elevated glucose. Hypertension was diagnosed as patients with systolic/diastolic pressures ≥ 140/90 mmHg, or taking antihypertensive drugs. T2DM was defined in accordance with the clinical classification and diagnosis of diabetes (24).

We also collected the following biochemical parameters: FPG; fasting plasma C-peptide; fasting plasma insulin; glycated hemoglobin (HbA1c); serum uric acid (SUA); creatinine; blood urea nitrogen (BUN); aminotransferase (ALT); aspartate aminotransferase (AST); γ-glutamyl transpeptidase (GGT); alkaline phosphatase (ALP); total bilirubin; direct bilirubin; indirect bilirubin; albumin; total cholesterol; triglycerides; HDL-C; low-density lipoprotein cholesterol (LDL-C)], and routine blood data pertaining to red blood cell (RBC), white blood cells (WBC) and platelet. Standard laboratory methods were used to carry out each of these biochemical tests. In addition, we also calculated homeostatic model assessment of insulin resistance (HOMA-IR) (insulin (mU/L) x FPG (mmol/L)/22.5) to indirectly assessed insulin resistance.

In addition, certain non-invasive fibrosis scores were computed using the relevant published formulas: APRI (AST to platelet ratio index) (13); FIB-4 (age, ALT, AST, platelet) (16); NFS (age, BMI, diabetes status, platelet, albumin) (15); BARD (BMI, AST/ALT ratio, T2DM) (14); Hepamet Fibrosis Score (HFS) was computed using a free web page: https://www.hepamet-fibrosis-score.eu/ (25)..

### Histopathological evaluation

Liver specimens were obtained, in the form of a wedge biopsy from the left lobe of the liver, by the surgeon performing the bariatric surgery. Liver tissue specimens were routinely formalin-fixed, paraffin-embedded and then stained with hematoxylin-eosin. The biopsy specimen was at least 10 mm long or not less than 10 portal tracts. All histological examinations were performed by the same experienced pathologist, blinded for clinical and laboratory data. Histopathological analysis was performed according to the steatosis, activity, and fibrosis (SAF) score (26). Fibrosis was graded as 0–4 stages (27): F0 = no fibrosis, F1 = perisinusoidal or periportal fibrosis, F2 = perisinusoidal and portal/periportal fibrosis, F3 = bridged fibrosis, and F4 = cirrhosis. NASH was defined as steatosis (5% of hepatocytes), hepatocellular ballooning and lobular inflammation. Significant liver fibrosis was defined as stage 2 fibrosis or above.

### Statistical analysis

Statistical analysis was performed using SPSS version 26.0 (SPSS Inc. Chicago, IL, USA), and MedCalc version 19.4.0 (Ostend, Belgium). Continuous data were presented as mean ± standard deviation (SD), whilst categorical data was given as a number (frequency or percentage). Pairwise comparisons of continuous data were performed using the t-test or Mann–Whitney test, whereas categorical data were compared using a chi-square test or Fisher’s exact test. Normality was assessed using the Kolmogorov-Smirnov test. To identify the predictive factors related to significant fibrosis, univariate logistic regression models were performed to identify each possible predictor. Then, multicollinearity was assessed using the variance inflation factor (VIF) method, with a VIF≥5 indicating the presence of multicollinearity, and no significant collinear variables were found. Finally, independent variables with statistically significant (P <0.05) were introduced into a multivariable logistic regression (backward selection method). An odds ratio (OR) with a 95% confidence interval (CI) was calculated.

In order to evaluate the performance of non-invasive scoring systems for detecting significant fibrosis, we calculated the area under the curve (AUC) of receiver operating characteristic curves (ROC) (AUROC), sensitivity, specificity, positive predictive value (PPV) and negative predictive value (NPV) along with their 95% CI. ROC curves were compared using the methods of Hanley & McNeil (28). Statistical significance was defined as a p<0.05.

## Results

### Clinical baseline characteristics

**Figure 1:**
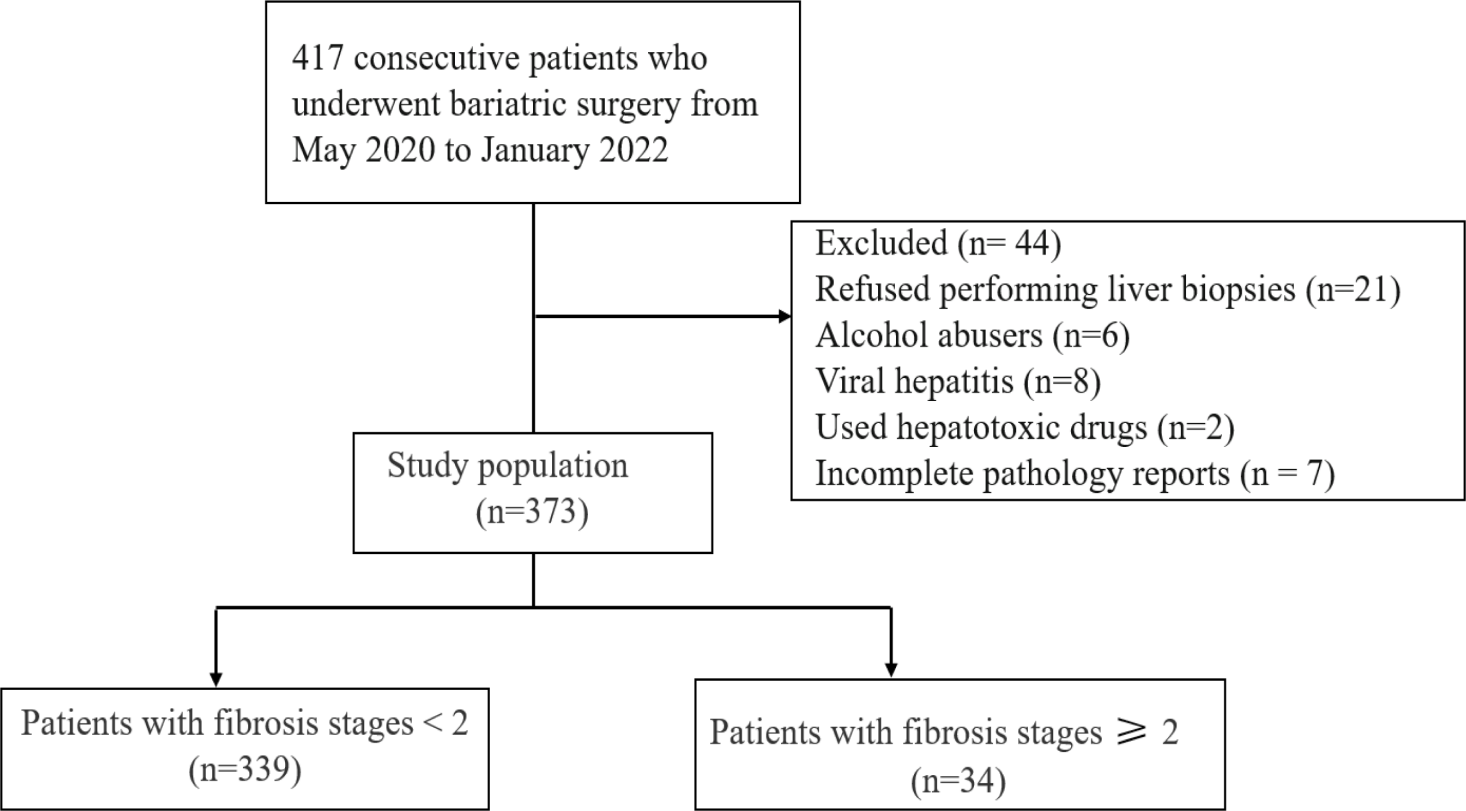
A flowchart illustrating the study approach.

Of the 417 consecutive patients who underwent bariatric surgery between May 2020 and January 2022, 44 patients exhibited criteria (detailed in methods) that meant they were excluded from our study. In total, 373 patients were recruited into this study, including 126 (33.7%) male patients and 247 (66.3%) female patients. Flow diagram of the study is shown in **Figure 1**.

The mean age and BMI of the study population were 30.9 ± 9.0 years and 39.4 ± 7.6 kg/m^2^, respectively. Patients with significant fibrosis tended to be older. They also exhibited: a higher prevalence of T2DM; higher levels of fasting plasma glucose, c-peptide, HbA1c, ALT, AST, GGT, and lower platelet counts compared to patients without significant fibrosis (p<0.05). When the non-invasive scoring systems were applied to our data, the results revealed that the significant fibrosis group had significantly higher scores than the patients assigned to the non-significant fibrosis group. A more detailed description of the study population is displayed in **Table 1**.

**Table 1:** Baseline characteristics of all patients and those with significant fibrosis and without significant fibrosis.

Variables	Total cohort (n=373)	Fibrosis stages F < 2 (n=339)	Fibrosis stages F ≥2 (n=34)	P value
Demographic characteristics				
Male, n (%)	126 (33.7%)	112 (33.0%)	14 (41.2%)	0.339
Age (years)	30.9 ± 9.0	30.4 ± 8.5	35.8 ± 12.0	0.018
Weight (kg)	109.5 ± 26.6	109.3 ± 26.7	111.3 ± 25.8	0.679
BMI (kg/m2)	39.4 ± 7.6	39.3 ± 7.6	40.3 ± 6.7	0.494
Waist circumference (cm)	120.1 ± 17.5	119.5 ± 17.6	125.4 ± 14.9	0.101
Hip circumference (cm)	123.2 ± 13.8	123.2 ± 13.9	123.3 ± 12.9	0.970
WHR	0.97 ± 0.09	0.97 ± 0.09	1.01 ± 0.10	0.006
Comorbidities				
Metabolic syndrome, n (%)	280 (75.1%)	252 (74.3%)	28 (82.4%)	0.303
Hypertension, (%)	106 (28.4%)	96 (28.3%)	10 (29.4%)	0.892
T2D, n (%)	124(33.2%)	102(30.1%)	22 (64.7%)	0.000
Laboratory data				
FPG (mmol/l)	6.6 ± 3.1	6.5 ± 2.8	8.3 ± 5.1	0.012
Insulin	23.6 ± 18.0	23.5 ± 18.3	24.7 ± 14.4	0.701
C-peptide	3.7 ± 1.6	3.6 ± 1.5	4.6 ± 2.1	0.010
HbA1c (%)	6.4 ± 1.6	6.3 ± 1.5	7.3 ± 1.9	0.000
HOMA-IR	7.1 ± 6.7	6.9 ± 6.6	8.7 ± 7.2	0.134
BUN (mmol/L)	4.5 ± 1.2	4.5 ± 1.7	4.8 ± 1.6	0.196
Creatinine (μmol/L)	62.5 ± 16.7	62.3 ± 16.0	66.6 ± 22.5	0.625
SUA(μmol/L)	450.7 ± 123.7	447.8 ± 122.9	479.1 ± 130.1	0.162
ALT (U/L)	59.1 ± 50.1	57.3 ± 50.5	76.8 ± 43.6	0.030
AST (U/L)	35.4 ± 27.0	33.3 ± 25.3	55.9 ± 34.2	0.000
AST/ALT	0.70 ± 0.27	0.69 ± 0.27	0.76 ± 0.25	0.152
GGT (U/L)	48.3 ± 38.8	45.8 ± 36.3	72.1 ± 53.7	0.001
ALP	83.2 ± 24.7	82.5 ± 23.9	91.1 ± 30.3	0.148
Total bilirubin (mmol/l)	11.7 ± 5.2	11.7 ± 5.0	12.4 ± 6.6	0.457
Direct bilirubin	3.3 ± 1.7	3.3 ± 1.7	3.5 ± 2.3	0.451
Indirect bilirubin	8.4 ± 3.8	8.4 ± 3.8	8.9 ± 4.6	0.513
Albumin, g/dL	42.4 ± 3.9	42.3 ± 3.2	43.2 ± 7.7	0.448
Total cholesterol (mg/dL)	5.2 ± 1.0	5.2 ± 1.0	5.0 ± 1.1	0.242
Triglycerides (mg/dL)	2.0 ± 1.9	2.0 ± 2.0	2.1 ± 0.9	0.853
HDL-c (mg/dL)	1.1 ± 0.2	1.1 ± 0.2	1.0 ± 0.2	0.211
LDL-c (mg/dL)	3.1 ± 0.8	3.1 ± 0.7	3.1 ± 0.9	0.954
WBC (10^12^/L)	8.4 ± 3.7	8.4 ± 3.7	8.4 ± 2.6	0.993
Platelets (109/L)	284.1 ± 67.0	286.6 ± 65.3	258.9 ± 78.9	0.042
Hepatic fibrosis index				
APRI	0.13 ± 0.11	0.12 ± 0.10	0.24 ± 0.18	0.000
FIB-4	0.56 ± 0.65	0.50 ± 0.25	1.35 ± 1.87	0.000
NFS	-2.2 ± 1.57	-2.33 ± 1.43	-0.96 ± 2.23	0.001
BARD	1.89 ± 0.98	1.82 ± 0.94	2.50 ± 1.21	0.001
HFS	0.05 ± 0.08	0.04 ± 0.05	0.18 ± 0.18	0.000

BMI, body mass index; WHR, waist to hip ratio; T2D, type 2 diabetes; FPG, fasting plasma glucose; HbA1c, glycated hemoglobin; HOMA-IR, Homeostatic model assessment of insulin resistance; BUN, blood urea nitrogen; SUA, serum uric acid; ALT, aminotransferase; AST, aspartate aminotransferase; GGT, γ-glutamyl transpeptidase; ALP, alkaline phosphatase; HDL-C, high-density lipoprotein cholesterol; LDL-C, low-density lipoprotein cholesterol; WBC, white blood cells; APRI, AST to Platelet Ratio Index; FIB-4, Fibrosis‐4 score; NFS, NAFLD fibrosis score; HFS, Hepamet fibrosis score.

### Prevalence of steatosis and significant fibrosis

**Figure 2:**
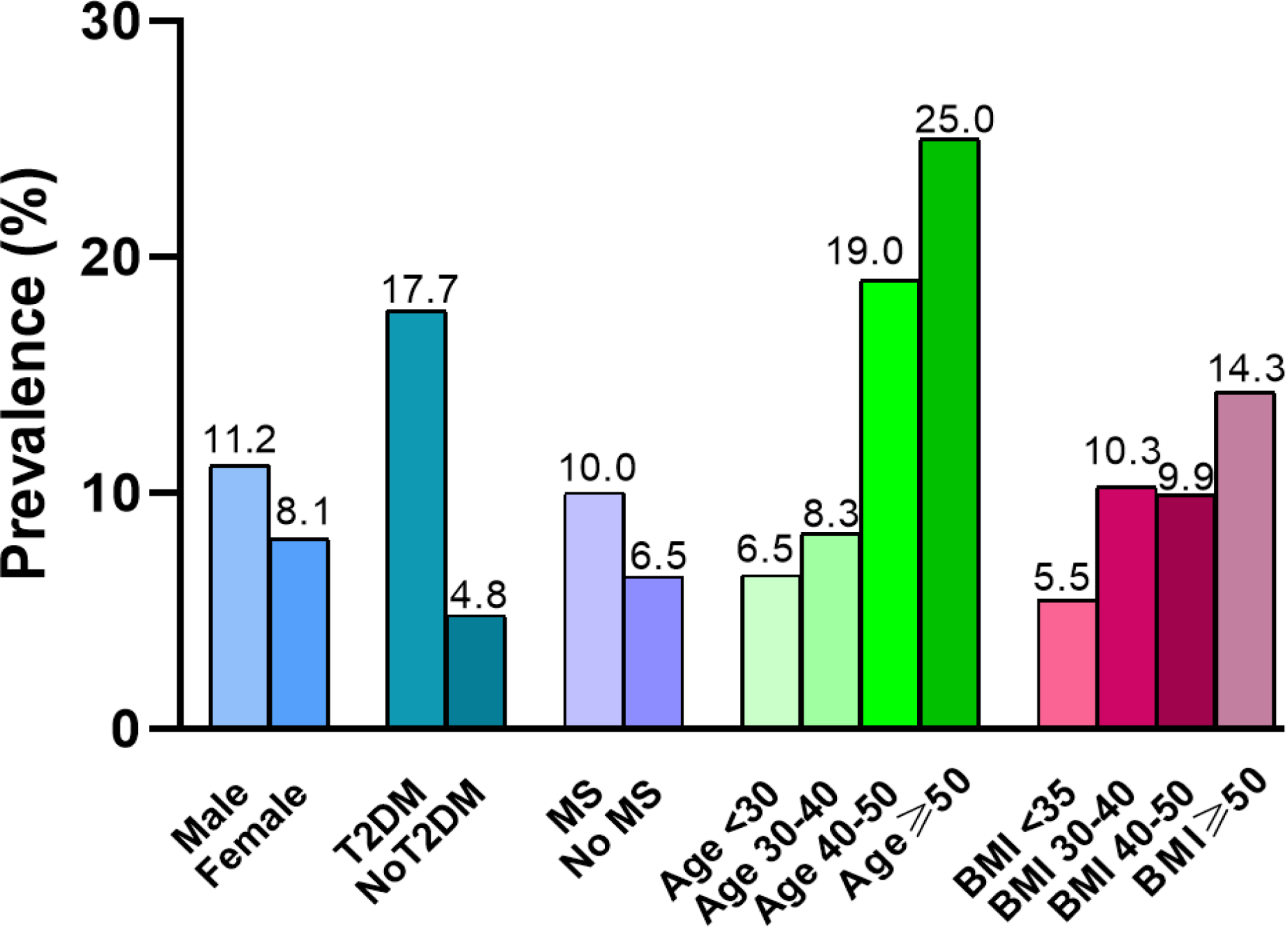
Prevalence of significant fibrosis stratified by gender, age, T2DM, MS and BMI.

Of those 373 patients, 89.0% (332/373) of patients fulfilled the NAFLD criteria and 68.9% met the NASH criteria. The overall prevalence of significant fibrosis (F≥2) was 9.1%. Our analysis showed that patients with T2DM have a significantly higher prevalence of significant fibrosis than those without T2DM (χ2 = 13.407, p=0.003). The prevalence of significant fibrosis increased significantly as age increased. We determined the frequency of fibrosis as 7.0% in individuals with age < 30 years rising to 25% in patients with an age ≥50 years (χ2 = 10.315, p=0.016). However, when patients were stratified according to gender, MS or BMI, there was no statistically significant correlation between the occurrence of significant fibrosis and any of these factors. In addition, we observed a 4.0% prevalence of advanced fibrosis (F≥3) and a 1.6% prevalence of cirrhosis in bariatric surgery patients. (**Table 2**, **Figure 2**).

**Table 2:** Liver biopsy characteristics of patients.

Liver histology	N (%)
Steatosis grade	
0	41(11.0%)
1	121(32.4%)
2	114 (30.6%)
3	97 (26.0%)
Lobular inflammation grade	
0	25 (6.7%)
1	165 (44.2%)
2	183 (49.1%)
3	0 (0)
Ballooning grade	
0	81(21.7%)
1	258 (69.2%)
2	34 (9.1%)
Fibrosis stage	
0	146 (39.1%)
1	193 (51.7%)
2	19 (5.1%)
3	9 (2.4%)
4	6 (1.6%)
NAFLD	332 (89.0%)
NASH	257 (68.9%)
Fibrosis (F≥1)	227 (60.9%)
Significant fibrosis (F≥2)	34 (9.1%)
Advanced fibrosis (F≥3)	16 (4.0%)

NAFLD, Non-alcoholic fatty liver disease; NASH, nonalcoholic steatohepatitis.

### Clinical predictors for significant fibrosis

To explore the predictive factors of significant fibrosis, clinical variables associated with significant fibrosis were evaluated using univariate analysis. Further analysis using a multivariable logistic regression model was performed based on variables with P < 0.05 in the univariate analysis (age, WHR, T2DM, FPG, c-peptide, HbA1c, AST, GGT, platelets). The results revealed that: age (OR], 1.06; 95% CI, 1.02-1.11, p=0.003); T2DM (OR, 2.62; 95% CI, 1.17-5.88, p=0.019); c- peptide (OR, 1.26; 95% CI, 1.03-1.55, p=0.025) and AST (OR, 1.02; 95% CI, 1.01-1.03, p=0.004) were detected as independent predictors of significant fibrosis. (**Table 3**)

**Table 3:** Univariate and multivariate logistic regression analyses were used to identify independent factors associated with significant fibrosis.

	Univariate analysis		Multivariate analysis	
	Crude OR (95% CI)	*P* value	Adjusted OR (95% CI)	*P* value
Male, n (%)	1.44 (0.70-2.95)	0.323	–	–
Age (years)	1.06 (1.02-1.10)	0.001	1.06 (1.02-1.11)	0.003
Weight (kg)	1.00 (0.99-1.02)	0.678	–	–
BMI (kg/m2)	1.02 (0.97-1.06)	0.494	–	–
Waist circumference (cm)	1.02 (0.99-1.04)	0.101	–	–
Hip circumference (cm)	1.00 (0.98-1.03)	0.970	–	–
WHR^#^	1.83 (1.20-2.80)	0.005	–	–
*Comorbidities*			–	–
Metabolic syndrome, n (%)	1.61 (0.65-4.02)	0.307	–	–
Hypertension, (%)	1.05 (0.48-2.28)	0.901	–	–
T2DM, n (%)	4.26 (2.03-8.93)	0.000	2.62 (1.17-5.88)	0.019
Laboratory data			–	–
FPG (mmol/l)	1.14 (1.05-1.24)	0.003	–	–
Insulin	1.00 (0.99-1.02)	0.701	–	–
C- peptide	1.33 (1.11-1.60)	0.002	1.26 (1.03-1.55)	0.025
HbA1c (%)	1.31 (1.11-1.56)	0.002		
HOMA-IR	1.03 (0.99-1.07)	0.144	–	–
BUN (mmol/L)	1.18 (0.92-1.53)	0.198	–	–
Creatinine (μmol/L)	1.01 (0.99-1.03)	0.449	–	–
SUA(μmol/L)	1.00 (1.00-1.01)	0.162	–	–
ALT (U/L)	1.01 (1.00-1.01)	0.038	–	–
AST (U/L)	1.02 (1.01-1.03)	0.000	1.02 (1.01-1.03)	0.004
AST/ALT	2.35 (0.73-7.60)	0.154		
GGT (U/L)	1.01 (1.01-1.02)	0.001	–	–
ALP	1.00 (1.00-1.03)	0.055	–	–
Total bilirubin (mmol/l)	1.03 (0.96-1.09)	0.456	–	–
Direct bilirubin	1.08 (0.89-1.30)	0.451	–	–
Indirect bilirubin	1.04 (0.96-1.13)	0.362	–	–
Albumin, g/dL	1.05 (0.97-1.13)	0.222	–	–
Total cholesterol (mg/dL)	0.80 (0.56-1.16)	0.242	–	–
Triglycerides (mg/dL)	1.02 (0.86-1.20)	0.853	–	–
HDL-c (mg/dL)	0.33 (0.06-1.86)	0.210	–	–
LDL-c (mg/dL)	0.99 (0.62-1.57)	0.954	–	–
WBC (10^12^/L)	0.99 (0.91-1.10)	0.993	–	–
Platelets (10^9^/L)	0.99 (0.98-1.00)	0.020	–	–

OR, odds ratio; CI, confidence interval. BMI, body mass index; WHR, waist to hip ratio; T2D, type 2 diabetes; FPG, fasting plasma glucose; HbA1c, glycated hemoglobin; HOMA-IR, Homeostatic model assessment of insulin resistance; BUN, blood urea nitrogen; SUA, serum uric acid; ALT, aminotransferase; AST, aspartate aminotransferase; GGT, γ-glutamyl transpeptidase; ALP, alkaline phosphatase; HDL-C, high-density lipoprotein cholesterol; LDL-C, low-density lipoprotein cholesterol; WBC, white blood cells.

#Per 0.1 increase 10.

### Comparison of non-invasive scoring systems

**Figure 3:**
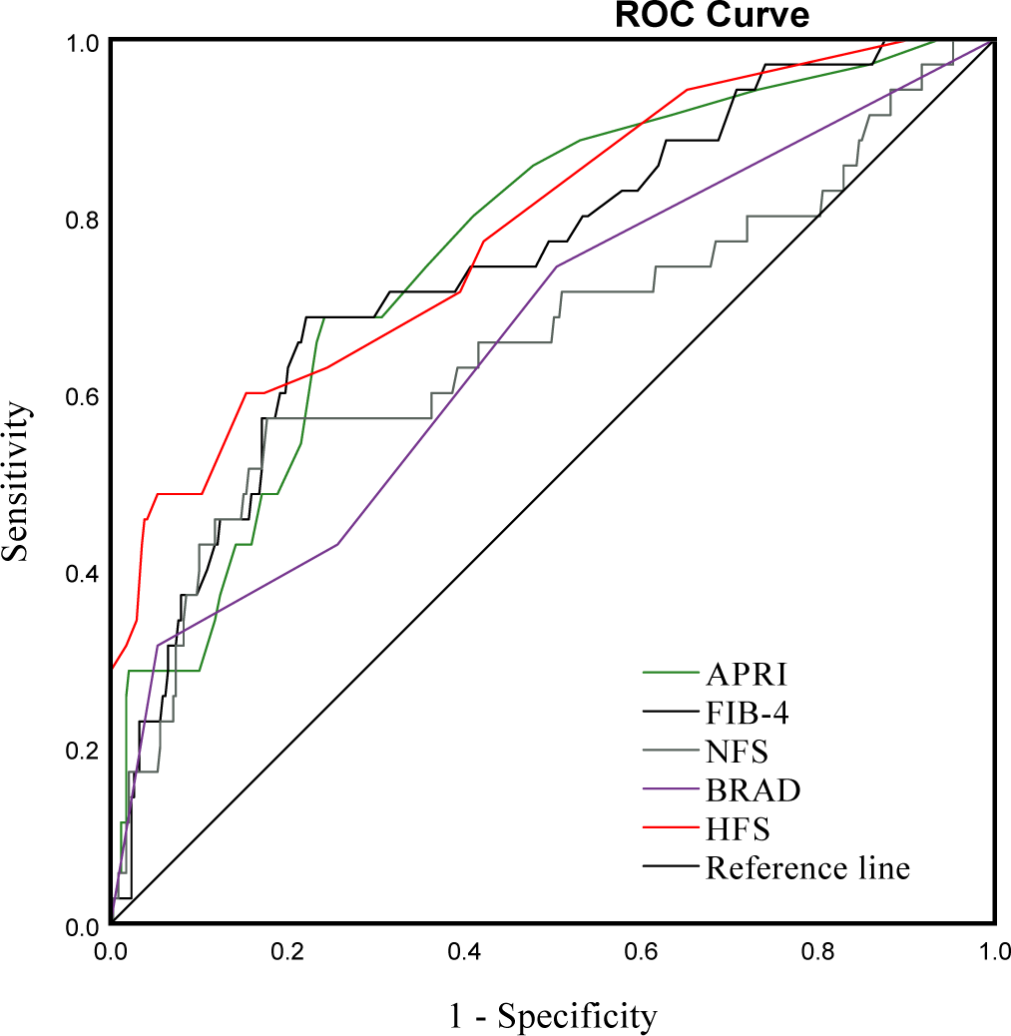
ROC curve for APRI, FIB-4, NFS, BARD and HFS in bariatric surgery patients with and without significant fibrosis.

To validate the reliability of non-invasive scoring algorithms for the diagnosis of significant fibrosis, we calculated the AUROC for the results of the five non-invasive scoring systems that were applied to our data. This yielded AUROC ranging from 0.652 to 0.781. The HFS had the best predictive performance, with an AUROC of 0.781, followed by the FIB-4 (0.745), APRI (0.759), NFS (0.657) and BARD (0.652) (**Figure 3**, **Table 4**). Pairwise comparison of the AUROC of different scoring systems demonstrated that there were significant differences between these non-invasive scoring systems, including APRI vs NFS, BRAD vs FIB-4, BRAD vs HFS, FIB-4 vs NFS and HFS vs NFS (all P < 0.05); while no significant differences between other non-invasive scoring systems were detected (all P > 0.05).

**Table 4:** Performance of the APRI, FIB-4, NFS, BARD and HFS for the detection of significant fibrosis.

	APRI	FIB-4	NFS	BARD	HFS
Cutoff value	0.43	0.46	0.38	0.24	0.43
AUC (95% CI)	0.759 (0.712-0.801)	0.745 (0.697-0.788)	0.657 (0.607-0.705)	0.652 (0.601-0700)	0.781 (0.735-0.822)
Sensitivity (95% CI)	67.6 (49.5-82.6)	67.7 (49.5-82.6)	55.9 (37.9-72.8)	29.4 (15.1-47.5)	58.8 (40.7-75.4)
Specificity (95% CI)	75.8 (70.9-80.3)	77.9 (73.1-82.2)	82.3 (77.8-86.2)	94.7 (91.7-96.8)	84.7 (80.4-88.3)
LR (+) (95% CI)	2.8 (2.1-3.8)	3.1 (2.3-4.3)	3.2 (2.2-4.6)	5.5 (2.8-11.0)	3.8 (2.6-5.6)
LR (-) (95% CI)	0.4 (0.3-0.7)	0.4 (0.3-0.7)	0.5 (0.4-0.8)	0.8 (0.6-0.9)	0.5 (0.3-0.7)
PPV (95% CI)	21.9 (17.2-27.4)	23.5 (18.4-29.4)	24.1 (17.8-31.6)	35.7 (21.8-52.5)	27.8 (20.9-35.9)
NPV (95% CI)	95.9 (93.5-97.4)	96.0 (93.6-97.5)	94.9 (92.7-96.5)	93.0 (91.5-94.3)	95.3 (93.2-96.8)

AUC, area under the curve; CI, confidence interval; APRI, AST to Platelet Ratio Index; FIB-4, Fibrosis‐4 score; NFS, NAFLD fibrosis score; HFS, Hepamet fibrosis score; LR likelihood ratio; PPV, positive predictive value; NPV, negative predictive value.

## Discussion

The presence of fibrosis in NAFLD patients affects clinical prognoses. NAFLD has got widespread attention in bariatric surgery patients, but there are still scant studies into the prevalence of significant fibrosis. For this reason, we first examined the prevalence and potential risk factors of significant fibrosis among Chinese bariatric surgery patients. Our results indicated an overall prevalence of significant fibrosis, advanced fibrosis and cirrhosis of 9.1%, 4.0% and 1.6%, respectively. Specifically, the odds of having significant fibrosis were independently associated with the presence of T2DM, increasing age, and elevated AST, c-peptide levels. Furthermore, we also validated the reliability of non-invasive scoring systems and found that APRI, FIB-4 and HFS showed appropriate AUROC (>0.70) for predicting significant fibrosis, but BRAD and NFS score revealed poorly predictive performance compared to the other scores.

Previous studies reported the prevalence of biopsy-proven NASH during bariatric surgery, ranging from 2.6% to 98% (4). Some potential explanations for the discrepancy in prevalence are different histological scoring systems, selection bias, race-based differences and variability of observations among pathologists. In this study, we observed 68.9% population had NASH and 60.9% had fibrosis, which was similar to those from Japan (77.5%) and Taiwan (71.3%) (4). In contrast, a study with 1000 patients who underwent routine liver biopsies during bariatric surgery showed the rate of NASH/fibrosis was only 14.3% (29). Another large-scale study including 2557 bariatric surgery patients also discovered that only 30.9% and 29.3% of individuals had NASH and fibrosis respectively (30). Obviously, our results were significantly higher than those from two studies (29, 30), as well as those from the USA (24.1-58.6%) and Australia (18.4-24.8%) (4). This discrepancy may be due to racial differences, as Asian populations (even individuals with relatively low BMI) have an elevated risk of metabolic disease due to differing body fat percentages and body composition (31). In addition, 9.1% of patients were found to have significant fibrosis, 4.0% had advanced fibrosis and 1.6% had cirrhosis. Our findings are in agreement with the study by Udelsman BV, which found that in a cohort of bariatric surgery patients, 7.8% had significant fibrosis and 3.6% had advanced fibrosis (30). However, another retrospective study of 330 patients undergoing routine liver biopsy during bariatric surgery showed an increased prevalence of significant fibrosis, although results for advanced fibrosis, and cirrhosis were more similar to our findings (20.9%, 4.2% and 1.5%, respectively) (32).

Significant fibrosis is an established risk factor for cirrhosis and overall mortality (33). Research has shown that advanced fibrosis can persist for many years despite substantial weight loss following bariatric surgery (34). Accordingly, the early identification of clinically significant fibrosis could potentially improve patient outcomes. Several independent predictors of advanced fibrosis have been reported in prior studies (9, 19, 25, 35), including increasing age, T2DM, HOMA-IR, hypertension, elevated AST, and decreased platelets. Of those predictors, T2DM is one of the most useful predictors of liver fibrosis. In this study, patients with T2DM have a higher prevalence of significant fibrosis than patients without T2DM. Glucose metabolism-related indicators, such as T2DM and c-peptide, were found to be strongly associated using multivariate logistic regression models. However, hypertension and MS were not accepted as predictors of significant fibrosis, in line with previous study (4, 36). In addition, our study found that increasing age and elevated AST were independently associated with significant fibrosis, as has been mentioned above predictors.

Current guidelines recommend utilizing non-invasive scoring systems to identify at-risk NASH or fibrosis (37). Among such non-invasive scoring systems, the APRI, FIB-4, BRAD and NFS are widely used to detect liver fibrosis (38). HFS was recently developed based on an international multicenter study with 2452 participants and provided superior performance to detect patients with advanced fibrosis with an AUROC of 0.85, the sensitivity of 74%, and specificity of 97.2%, when compared with the FIB-4 and NFS systems (25). Another international multicenter retrospective study of 379 biopsy-proven NAFLD patients showed HFS and FIB-4 had higher AUROC for identifying significant fibrosis (0.744 and 0.725, respectively) than that of the no NFS, but no statistical differences were found between HFS and FIB-4 AUROC (39). Similarly, a retrospective study including 222 patients with biopsy-proven NAFLD demonstrated that the HFS(AUROC,0.758) was marginally less superior than FIB-4(AUROC,0.796) in detecting advanced fibrosis (40). In this study, APRI, FIB-4 and HFS all showed sufficient prediction accuracy (all AUROC ≥0.70), but there were no significant differences between APRI, FIB-4 and HFS AUROC. Compared to other scoring systems, BRAD and NFS scores did not exhibit satisfactory diagnostic performance in detecting significant fibrosis. In this prospective derivation and global validation study, the accuracies of BRAD and NFS for predicting significant fibrosis were 0.58 (0.54–0.62) and 0.66 (0.62–0.70), respectively (7). In the study by Zambrano-Huailla R et al, NFS was unable to effectively detect significant fibrosis in patients with NAFLD, with an AUROC of 0.581 (39). Thus, the role of the BRAD and NFS in predicting significant fibrosis in bariatric surgery patients should be further explored. Based on the current results, we can use non-invasive scores (APRI, FIB-4 and HFS) to monitor these patients with fibrosis closely. (Reviewer #2)

The strength of our study is that it was the first to prospectively evaluate the prevalence and clinical predictors of biopsy-confirmed significant fibrosis among Chinese bariatric surgery patients. However, we acknowledged there were several limitations in the current study. Firstly, this was a single-center cross-section study, limiting our study’s generalizability. Secondly, the biopsy samples were only from the left lobe of the liver, which may lead to misclassification of liver fibrosis severity as, in terms of histology, severity varies depending on the specific area of the liver being biopsied (41). Thirdly, some drugs, such as lipid-lowering drugs, antihypertensive drugs and antidiabetic drugs, may influence the results. Finally, we could not evaluate the application of this test in bariatric patients, because our hospital lacked “FibroScan”. (**Reviewer #1)** Therefore, multicenter studies with larger sample sizes should be undertaken to better evaluate the prevalence of fibrosis and its predictive factors in Chinese bariatric surgery patients.

## Conclusions

Our study showed more than two-thirds of bariatric surgery patients had NASH, and the prevalence of significant fibrosis was high. Risk factors for significant fibrosis include increasing age, presence of T2DM, elevated AST and c-peptide levels. Non-invasive models (including APRI, FIB-4 and HFS) can help clinicians to identify significant liver fibrosis in bariatric surgery patients. Further multicenter studies with larger sample sizes on liver fibrosis are warranted in bariatric surgery patients.

## Data availability statement

The raw data supporting the conclusions of this article will be made available by the authors, without undue reservation.

## Ethics statement

The studies involving human participants were reviewed and approved by the Ethics Research Committee of the First Affiliated Hospital of Jinan University (2019-024). The patients/participants provided their written informed consent to participate in this study.

## Author contributions

YS and WC: conceptualization and writing-original draft preparation. YS, SD, and WC: methodology, data curation, and formal analysis. CW and ZD: resources, supervision, and project administration. CW, ZD, and WC: writing-review and editing. All authors contributed to the article and approved the submitted version.
